# From Corona Virus to Corona Crisis: The Value of An Analytical and Geographical Understanding of Crisis

**DOI:** 10.1111/tesg.12428

**Published:** 2020-06-09

**Authors:** Verena Brinks, Oliver Ibert

**Affiliations:** ^1^ Johannes Gutenberg‐Universität Mainz Geographisches Institut Johann‐Joachim‐Becher‐Weg 21 Mainz 55128 Germany; ^2^ Leibniz Institute for Research on Society and Space (IRS) Flakenstr. 29‐31 Erkner 15537 Germany; ^3^ Brandenburg University of Technology Cottbus‐Senftenberg Platz der Deutschen Einheit 1 Cottbus 03046 Germany

**Keywords:** crisis definition, crisis management, geography of crisis, transboundary crisis, media reports, TPSN framework, COVID‐19

## Abstract

The term ‘crisis’ is omnipresent. The current corona virus pandemic is perceived as the most recent example. However, the notion of crisis is increasingly deployed as a signifier of relevance, rather than as an analytical concept. Moreover, human geography has so far little contributed to the interdisciplinary crisis research field which is fixated on the temporal aspects of crisis but neglects its spatiality. Against this background, the first aim of the paper is to demonstrate the value of thinking about crisis analytically. Therefore, we introduce theoretical knowledge developed within a recently emerging literature on crisis management. Second, we demonstrate the relevance of including geographical thinking into crisis research more systematically. Based on the TPSN‐framework by Jessop *et al*., we illustrate spatial dimensions of the ‘corona crisis’, its perception and handling in Germany. The empirical references are based on media reports.

## Introduction

The spread of the coronavirus has turned into a crisis. This finding is hardly surprising and the majority of readers will agree. But when and how did it turn into a crisis? This question is much more difficult to answer, mainly due to the fact that the term ‘crisis’ is anything but easy to grasp. It is omnipresent and frequently used in very different contexts. The term is, for instance, used to signify the enhanced relevance of the respective research. As a consequence, the notion of crisis is mainly deployed intuitively, rather than analytically.

Similar to other disciplines, most geographical contributions related to crisis dynamics are driven by an empirical phenomenon, which is framed as being in crisis. In economic geography, the ‘financial crisis’ has received particular attention and geographers have made significant contributions by exploring the manifold spatial references of this global phenomenon (e.g. Aalbers 2009; Martin 2011).

Within the geographic discipline, diagnoses of ‘crisis’ are often associated with neoliberalism and capitalism apparently producing manifold social, economic and political stresses (Jones & Ward 2002; Larner 2011). Particularly, geographers in Marxist tradition (most prominently represented by David Harvey) deploy crises as an inherent and recurring feature of capitalism; or to cite Harvey (2011, p. 11): ‘*capital never solves its crisis tendencies*’ (emphasis in orig.). In addition, climate change, rising religious fundamentalism and newly emerging economic powers outside the traditional industrial centres pose challenges of truly global scope that invoke crises in all parts of the world (Larner 2011). This literature emphasises that we live in times of crises and doubtlessly provide important insights on the social, economic and political configurations that contribute to crisis diagnosis.

However, so far little attention has been paid to specify the nature of ‘crisis’ itself as an exceptional and stressful human experience. If we accept the diagnosis that we live in times of crises, it becomes more important than ever to develop a more profound understanding of crisis as a particular context for action and a possibility for intervention. In this paper, we therefore propose a conceptual shift from the structural conditions that cause crises to an actor‐centric approach focused on the practical consequences of crisis for individual and collective agency. We introduce theoretical knowledge developed within a recently emerging, interdisciplinary literature on crisis management. Moreover, we illustrate this analytical understanding with reference to the recent ‘corona crisis’ to connect abstract ideas on the general characteristics of crises with empirical observations.

The term crisis is ripe with temporal implications. These temporal aspects predominate in the crisis management literature. What lacks so far, however, is a systematic conceptualisation of the spatial aspects of crisis. While the crisis management literature does use spatial categories, such as epicentres, distance, scaling or territories, it lacks a systematic approach to integrate spatial imaginations into theories and practices of crisis management. Social and economic geographers could thus contribute to the inter‐disciplinary discourse by integrating the spatial dimension into the conceptualisation of crisis. In this paper, we set out to suggest a conceptualisation of the ‘geography of crisis’ informed by social and economic geography.

The empirical material presented in this paper stems from different media sources. It has to be mentioned that this paper does not draw on an already fully‐elaborated or finalised media analysis but is inevitably provisional and selective due to the highly dynamic development at the time this paper has been written. The analytical and conceptual thoughts presented here are based on current research on crises (Brinks & Ibert 2020). Within different research contexts, we analysed literature on crisis (management) and also benefited from empirical insights collected in interviews with crisis experts and own participation in interdisciplinary workshops on crisis and crisis management.

The paper is subdivided into two main parts. First, the subsequent chapter introduces our definition of crisis based on literature from the interdisciplinary practice of crisis management and social scientific crisis research. Second, we outline a geographical perspective on crisis, by exploring different dimensions of the spatiality of crisis. We use the TPSN approach as suggested by Jessop *et al*. (2008) to systematise our observations. The paper concludes by highlighting the added value of a geographical approach.

## *Uncertainty, Urgency, Threat*: Insights From Crisis Research

A crisis is related to, yet distinct from other terms, such as ‘problem’. A problem denotes a gap between an observed condition and a desired condition (Rittel & Webber 1973). Such a gap is present in every crisis as well, for example, the gap between the fast‐growing numbers of people who became infected with the corona virus and the general desire that the population should be healthy. Yet, such a gap is not a sufficient condition for a crisis.

In order to talk of a crisis, a few more ingredients are necessary: uncertainty, urgency and threat (Boin & ‘t Hart 2007). *Uncertainty* denotes ‘that we cannot predict or foresee what will happen when acting or not acting’ (Aspers 2018, p. 133). In the corona case, uncertainty is caused by a lack of knowledge (e.g. about the ways of infections, dark figures of a‐symptomatic cases), ambiguous signals (e.g. unspecific symptoms), a lack of viable means to counter the epidemic (e.g. the absence of an effective medicine and vaccination) and undetermined timeframes (e.g. when will a vaccination be available), to mention only few. The second ingredient, *urgency*, refers to the necessity to act, despite high degrees of uncertainty. In crisis, inactivity and non‐decision are no options as they will only exacerbate the serious situation. Yet, as acting has to take place under conditions of uncertainty, routines are no longer available and action has a strongly improvisational or experimental character (Boin & Rhinard 2008; Milstein 2015). The last ingredient to crisis is an *existential threat* of highly valued societal assets. The corona pandemic does not only threaten the health and lives of wide parts of the population, but also imperils economic interests and core institutions of the political order. Due to the underlying fundamental uncertainty, ‘the emotional response to crisis is not fear (such as fear from fire) but existential angst, which has no identifiable object that could offer a grip for a learnt response’ (Kornberger *et al*. 2020, p. 242).

In addition to these fundamental characteristics of crisis it is important to unpack the term a bit further. It is important to understand how crisis becomes enacted in practice. A crisis as an empirical observation cannot be deduced directly from the underlying societal conditions. Rather similar, objectively measurable conditions (like unemployment rates, levels of distrust in political institutions) sometimes entail crisis diagnosis, and sometimes do not. Sometimes relatively unimportant issues are treated as a crisis (the ‘Brent Spar’ controversy is a widely cited example of an escalating risk communication; Löfstedt & Renn 1997), while even the most alarming scientific reports about climate change are not sufficient to mobilise a collective sense of urgency. What all crises share in common, thus, is not only a severe problem, but a shared perception of uncertainty, threat and urgency around that problem.

The key importance of perceptions can also be found in the corona case. In our observation of the public discourse in Germany, at the beginning of 2020 the government as many others in the Western hemisphere looked at the early epicentre of the pandemic, the Wuhan region in China, ‘with a combination of fascination and fear’ but without any sense of urgency or immediate threat until new information about corona infections in Europe emerged (Boin *et al*. 2020). Not earlier than 26 February 2020, we noticed a shift from ‘corona *epidemic*’ to ‘corona *crisis*’ in the German speaking debate for the first time in an article published in the online portal of the German newspaper *Der Spiegel*. Two days earlier, in many parts of Germany, carnival was celebrated on the streets – a mass meeting with thousands of people standing close to each other. From 16 March onwards, all public events of major size were prohibited, schools were closed across all Federal States in Germany and a few days later restaurants, production plants and retail shops followed. It was a matter of days, during which the publicly shared framing of the situation has changed fundamentally. Typically, at some stage in the public ‘framing contest’ that takes place in advance of a crisis, the public opinion transcends an invisible ‘tipping point’ beyond which a problematic situation turns into a crisis (Boin *et al*. 2009). However, this tipping point can only be noticed *ex post*, while it is impossible to determine it in advance. For most participants, thus, crisis comes unexpected.

Crisis is not only a matter of perception; it also unfolds performative qualities. Here, performative means that the crisis diagnosis is not a mere description of the state of reality Rather, a crisis diagnosis changes reality and therefore contributes to the enactment of crisis: ‘If individuals (and the media) define a situation as a crisis, it is a crisis in its consequences’ (Rosenthal & Kouzmin 1997, p. 286). For decision‐makers, once in place, the crisis immediately ascends the first place of the agenda. Due to the performative qualities, the crisis unfolds its dynamics irrespective of subjective interpretations or experiences. For individual decision‐makers, it is for instance no longer possible to ignore the crisis, or, if one tries, like Donald Trump did until the first weeks of March, it happens at immense political and economic costs. For professional crisis managers, the declaration of a crisis has very practical and robust consequences. They perceive a crisis as an effective ‘coping structure’ (term used in crisis management practice jargon) societies and organisations have to prioritise a certain topic and to mobilise resources to address a problem. Crisis diagnoses emerge in multi‐stakeholder constellations. Some stakeholders even support the escalation of a crisis or reframe the crisis diagnosis in ways that exert pressure on organisations or states. Crisis diagnoses thus are contested and the framing is subject to controversy in the public debate (Boin *et al*. 2009).

In contrast to the term ‘catastrophe’, the term crisis highlights that despite existential threats, it is not yet too late to prevent the disaster (Boin & ‘t Hart 2007). In medicine, crisis marks the decisive phase in the course of an illness in which a positive or negative outcome is still possible (Ricœur 1988). Crisis, in other words, is strongly associated with the idea of an open future (Kornberger *et al*. 2020) that can be created through individual or collective agency. In the current crisis, the first discussions emerge about the potential long‐term structural effects of the corona crisis. For instance, visionaries from Silicon Valley highlight the enhanced possibilities to establish new practices of remote digital learning and work (Thrun 2020). At the same time, warning voices (Sennett 2020) point at potentially problematic long‐term effects of lockdown policies and the increased use of surveillance technologies on the human rights situation and vulnerable democratic institutions in weak democracies.

When we think of or undertake research on crisis, we should also be aware of one additional observation. As mentioned above, decisions in crisis have to be made while the present is uncertain and the future is open (Kornberger *et al*. 2020). Under such conditions, action does not take place within a given frame of meaning. Rather, in crisis participants are forced to learn by interpreting the situation tentatively while acting on it. Therefore, crises are usually perceived twice. In a first loop, participants encounter a critical turn in the course of events surprisingly. They experience an open‐ended phase of chaos and escalation during which they struggle to regain control while action and sensemaking remain incompletely connected. In contrast to the abrupt beginning, the end of the acute crisis comes much more gradually. As a first step toward a (new) normality, after having responded to the challenges, participants eventually perceive a slowing down in the dynamic of escalation and try out new interpretations of the situation. However, against the background of the previously experienced uncertainty, participants tend to distrust this new stability. They remain unsure, how far their explanations will hold and whether or not the absence of another surprising turn is just a pause in the course of escalation or already a (re)turn to (new) normality. The ultimate end of the crisis, however, has to be ‘declared’ by decision‐makers, which is another performative act.

In a second loop, the course of events that led to the acute crisis is reconstructed *ex post* in the light of the newly established sense and certainty. As an interpretative act of sensemaking, the starting point and the end of the second loop are not fixed and can never be defined in advance. According to Weick (1988, p. 306), sensemaking is enacted since ‘parts of what the explorer discovers retrospectively are consequences of his own making’. By the very process of acting in crisis, a rising stream of new information and experiences have to be included in the sensemaking process. Thus, the second loop always starts after the first loop but usually at an early stage in the crisis course.

In this second loop, the crisis is deliberately embedded in the classical phase model encompassing the phases of pre‐crisis, acute crisis and post‐crisis (e.g. Fink 2002), while the boundaries between the phases are still in motion. During sensemaking the considered timeframe is expanded both, into the past and the future. When reflecting on the pre‐crisis phase, the focus is on weak warning signals that have been neglected beforehand or wrong decisions that contributed to an escalation of events. The post‐crisis phase, in contrast, provides the (oftentimes missed) opportunity to learn from the crisis (Birkland *et al*. 2009). Once the crisis is overcome, time is ripe to reiterate the acute crisis several times in order to get a detailed understanding of the sequential order of actions. Of course, the acute crisis itself cannot be repeated. Yet, the pre‐ and post‐crisis phases cannot emerge without the experiences made during the acute crisis.

The awareness of these two loops of crisis experience is helpful to keep in mind for the corona crisis. While writing this paper, we are still witnessing the escalation of events and the tentative form of sensemaking while acting on the situation. Yet, we can already discover first signs of time expansion. Presently the public debate has already turned towards the past by discovering early warning signs that have previously been ignored. For example, a paper published in March 2019, in which the authors warned against (at that time) future outbreaks of a corona virus caused by cross‐species transmission (Fan *et al*. 2019) has received broader attention in the last weeks, that is one year after publication. In China and in Italy suspicious accumulations of pneumonia cases attract the attention of epidemiologists aiming at reconstructing the outbreak. At about the same time, governments around the world start to plan for the future. They design graduated schemes back to normality, envisioning the possible ends of the crisis and speculating about new post‐corona normalities.

Finally, the severity of a crisis is widely associated with its perceived scope. The scope of the crisis describes the degree to which the perceived escalation of problematic events can be contained within separable units of society. Critical events are much more likely perceived as severe crises, the more they ‘spill over’ (Bundy *et al*. 2017) existing boundaries. On the territorial level, ‘transboundary crises’ (Boin & Rhinard 2008) have an inter‐regional or even an inter‐national character. Transboundary also denotes the overstepping of institutionalised boundaries, for example, the vertical sectoral responsibilities of political or administrative bodies (Boin & Rhinard 2008). A true sense of crisis tends to emerge if multiple boundaries are overstepped and causes and effects of a crisis spill over from one compartment into the other.

The scope of a crisis is also dependent on the sources of uncertainty. Sometimes, these sources are clearly external, for instance, an earthquake or a cyber‐attack. Such external events can unfold disruptive qualities, yet they are usually easier to manage, as no decision‐maker can be directly blamed for them. It thus seems sufficient to manage their negative consequences before returning back to old normality. More difficult are crises that are driven by internal sources. For instance, the structural crisis of a whole industry to a wide degree is caused by the insufficient strategic capabilities of the core decision‐makers. Here, the crisis is interpreted as a ‘brutal audit’ (Orton & O’Grady 2016) that unveils the lack of foresight and understanding of decision‐makers. Crises caused by internal factors enact a much higher degree of uncertainty, as any framing of the problem goes hand in hand with blaming of responsible persons or organisational units (Boin *et al*. 2009). Of course, in practice, it is difficult to clearly separate internal from external sources of uncertainty, as often critical external events raise the awareness of internal deficiencies.

In the case of the corona pandemic, the crisis fulfils the character of a ‘transboundary crisis’ (Boin & Rhinard 2008) in an almost ideal‐typical sense. The spread of the virus is no longer restricted to any geographically confined territory, vertical segments of society or particular societal layers. Rather, within a few months, the virus is present almost everywhere on the globe, justifying the WHO‐classification as a ‘pandemic’. Further, it affects several societal systems, most crucially the health services, but beyond that also has severe spill‐over effects to almost every economic sector, a wide range of institutions of political order and all parts of society. The tendency to transgress boundaries also makes the corona crisis particularly threatening. While the origin of the crisis is external to society, the corona pandemic can be seen as a brutal stress test that unveils internal dysfunctionalities in national health systems, social security programmes or value chains.

## A Geographical Perspective on (Corona) Crisis

Even though the geographical dimensions of crisis are recognised by some crisis scholars, a systematic and theoretically‐guided analysis of the spatiality of crisis has not yet been advanced in this field. Such a systematic exploration, we argue, is a possible contribution of economic and social geography to social scientific crisis research. The agenda we suggest here is thus a bit different from previous geographical studies that use the term crisis prominently to signify they are dealing with severe problems within specific empirical fields, like, for instance, the bursting of financial bubbles in mortgage and real estate markets (e.g. Aalbers 2009) or emergency practices in humanitarian aid (e.g. Fredriksen 2014).

We suggest the use of the TPSN framework (territory, place, scale, network), as developed by Jessop *et al*. (2008) to explore the geography of crisis. According to Gailing *et al*. (2019, p. 15) it provides a useful heuristic that can be flexibly applied to diverse empirical fields ‘to allow for a synoptic perspective on this field’. At the same time, the authors also warn that TPSN should not be mistaken as a ‘complete answer to everything’ (Gailing *et al*. 2019, p. 15), as it lacks the necessary, field‐specific theoretical terminology. Hence, they argue that TPSN needs to be complemented with the respective theoretical terminology to unfold its full explanatory potential. For our agenda, the absence of theoretical assumptions in the TPSN‐heuristic is an advantage. Crisis, as we understand it, is not an ‘empirical field’ in the sense of Gailing *et al*. (2019), but rather a conceptual endeavour to advance a general understanding of practices and dynamics prevailing in situations of uncertainty, threat and urgency. In the following paragraph, we thus use theoretical claims from social scientific crisis research and combine it with spatial dimensions as suggested in the TPSN heuristic in order to delve deeper into the so far underdeveloped spatial aspects of crisis theory. In the following, some starting points for such an investigation are indicated by referring to the corona crisis as one illustrative empirical field (Table 1).

**Table 1:** Spatial categories, illustrated by the corona crisis (own table; based on Jessop *et al*. 2008).

Space dimension	Examples from Corona crisis
Territory	Portrayal of outbreak according to territorial entities
	Activation of territorially‐bound resources
	‘First case’ inside or outside a territory
Place	Emergence of places of crisis such as supermarkets
	‘Epicentre’ and ‘super‐spreader’ locations
Scale	Assignment of responsibility
	Inter‐national organisations such as the WHO
Network	Expert communities
	‘#Flattenthecurve’

Even though crises increasingly cross territorial boundaries, the *territorial dimension* remains particularly important. The corona crisis produces countless cartographic visualisations documenting the spread of the pandemic. The number of infections announced by the Johns Hopkins University (2020) has become an internationally much‐cited data source for tracing the dynamic development of the spread as well as regional differences worldwide. The total number of confirmed cases worldwide is presented on the national level. Recently, a further map demonstrating the intensity of the outbreak in US counties has been launched by the university (Johns Hopkins University 2020). The territorial representation of the corona crisis is largely caused by the report system of public agencies which are bound to territorial units. Likewise, many institutional crisis responses, such as the official declaration of an emergency situation, are bound to territories. However, territory affects crisis even beyond administrative responsibilities. The crossing of a territorial boundary, for instance, frequently cause shifts in the perception of crises as being more threatening (since the perceived distance to crisis declines) and escalating (fear of losing control). ‘Patient 1’ as the first documented case in a certain territory is well reported as well as the first case of Covid‐19 outside of China on 13 January. Manifold media reports refer to ‘first cases’ or ‘first deaths’ inside or outside a specific territorial unit.

Some *places* are more affected by crisis than others (see Aalbers 2009 for the financial crisis). Some crises culminate in a single epicentre. A school shooting creates such a mono‐centric geography and ‘place renewal’ can be an adequate way for crisis recovery (Wombacher *et al*. 2018). More typically, however, crises unfold complex, multi‐local geographies. In the case of the corona pandemic, we can already identify several symbolically charged places. Above all, the Huanan Seafood market in Wuhan has been reported as the point of origin of the outbreak. Related to that, the use of the term ‘Wuhan Virus’ by the US government can be conceived as a framing and blaming strategy (Boin *et al*. 2009) through spatial dissociation and association (Ibert *et al*. 2019). Further places, such as the notorious après ski bars in Ischgl in Austria, or the football stadium in Milan have become spots of investigation as potential ‘super spreader’ locations from where the virus disseminated across Europe (Merlot 2020). Surprisingly, supermarkets have emerged as relevant places of the corona crisis. As places of food provision, in times of lockdown these facilities have transformed rapidly into critical infrastructures equipped with additional safety precautions. In contrast, hospitals represent classical institutions of crisis response. Yet, when becoming activated for this crisis, their regular safety standards needed to be adapted to the particular challenges of the corona pandemic.

The notion of *scale* is closely related to spatial hierarchies (Jessop *et al*. 2008). It is a particularly important dimension in crises when it comes to negotiation of responsibility and coordination of action (which scale is the right one to (re)act on crises?). The corona crisis provides a vivid example here. In Germany, for instance, the corona crisis induced a discussion of the federal constitution. Where in other states, the national governments decided about the closing of retail stores, etc., the national government in Germany is not authorised to decide about such measures since infection protection is situated at the federal state‐level *(Bundesländer)* (Leitlein & Schuler 2020). Moreover, the health authorities, which report about confirmed corona infections and are authorised to impose measures such as quarantine, are based on the level of administrative districts *(Landkreis)* or district‐free cities in Germany. Located at an inter‐national scale, the WHO receives particular attention in these days. Though not authorised to impose measures, the WHO has an important function in terms of policy recommendation. The WHO’s declaration of the COVID‐19 outbreak being a ‘pandemic’ on 11 March can be interpreted as a means justifying considerable state interference with fundamental rights.

The *network* perspective on crisis focuses on the relations between nodes (of every kind). It can be enriched by deploying the concept of ‘relational proximity’ (Gertler 2008) and the function of medical experts in the corona crisis. Medical professionals such as epidemiologists and virologists currently receive particular attention as policy advisors. They are embedded in trans‐local professional communities. They share knowledge about the corona virus internationally, for instance, through rapid publication practices in academic journals (see for instance *The* *Lancet*). Members of these professional communities are characterised by relational proximity, which means that based on a shared repertoire of practices and similar expertise they are able to collaborate closely even across physical distance. Another example of the network dimension are social media having an enormous relevance in the corona crisis in terms of establishing a common understanding of the situation and sharing (similar) experiences across distance. Calls such as ‘Flatten the curve’ or ‘Stay at home’ went viral online and contributed to a shared perception of the corona crisis even when the locations and individual concerns with the corona virus are different.

As Jessop *et al*. (2008) argue, the empirical reality cannot be separated into the categories territory, place, scale and network. Rather, the dimensions are interwoven in ‘sociospatial relations’. Similarly, Gailing *et al*. (2019) find typical nexuses between several dimensions when studying empirical cases from the German *Energiewende*. The following sub‐sections aim at providing some examples of such interactions between spatial dimensions in the corona crisis – importantly, without any claim of completeness and admittedly presented in a rather sketchy and unsystematic fashion. At the present state, it would be an impossible endeavour to outline all spatial relations, too dynamic is the escalation in the course of events. Therefore, we focus on three nexuses that can be detected in prominent public discourses to demonstrate the principle of our approach.

### Network‐place: topologies of interconnected places

As mentioned earlier, supermarkets have turned into strategic places in the fight against the pandemic across the globe. During the past few weeks, we witness a gradual reshaping of their physical setup and practices of staff to accommodate these places to the new requirements of ‘social distancing’ while maintaining a high turn‐over of people. Items from hospital environments, like surgical masks and gloves have been transferred to supermarkets in order to protect staff and clients. Planes of acrylic glass have been fixed at checkout counters to minimise the physical contact between cashiers and customers and tapes attached on the floor remind shoppers to hold minimum distance. At the same time, familiar items, such as customer divider bars, loyalty cards or cash money have been banned from some supermarkets as they are now reinterpreted as potential carriers of the virus.

However, it would be inaccurate to primarily conceive supermarkets as singular places. Most supermarkets are not single‐owned stores but rather branch stores belonging to chains of multi‐national retail chains. Of course, supermarkets are places, though places that belong to wider networks operated and orchestrated by grand retailers. Supermarkets, in other words, are part of networks of practices (Brown & Duguid 2001). The concrete local practices and settings are thus not idiosyncratic, but depend strongly on the affiliation to a certain retail chain. Moreover, these practices might vary slightly from chain to chain while they are made similar from place to place through standards orchestrated through the respective networks. A similar topological perspective on crisis has been elaborated by Fredriksen (2014). The author focuses on emergency infrastructure which is used in different humanitarian crises. According to the author, emergency tents as material objects, which have constantly been developed further after crisis experiences, represent ‘lessons learned’ from different crises. Moreover, since they are highly mobile and used at different sites affected by crisis, the places resemble one another and thus become nodes in a ‘network topology’ of crises (Fredriksen 2014).

In a longer timeframe, experiences gained in supermarkets will most probably turn out to be extremely important for all kinds of retail stores. As soon as legislation will step by step relax the regulations on social distancing, the network of practice will most likely expand from the realm of supermarket(s) (chains) to other retailers, for instance in fashion retail or book stores.

### Scale‐territory: negotiation of crisis governance

The connection of scale and territory is obvious in crisis settings since public crisis response strategies are usually immediately connected to territorial units. *Scaling* in the sense of deciding which level is the most effective one for coping with crises is a key question in crisis management (e.g. Boin *et al*. 2005). The different levels usually present territorial units where the smallest level is always fully integrated in the next larger level (municipal level, national scale, European scale, etc.). This leads to the key issue of *coordination* in crises. A certain threatening situation has to be assigned to a specific scale, responsible for crisis response. These responsibilities are usually determined beforehand. In the corona crisis, the formal assignment of authority in epidemic events (as mentioned, the federal states *(Bundesländer)* are responsible instead of the national government in Germany) is now critically eyed and political efforts have been started to change the respective law in order to allow the upscaling of competencies to the national level in such crises (Waschinsky 2020). At the same time, local hubs of the outbreak are intensively investigated such as the district of Heinsberg in the federal state of North Rhine‐Westphalia in Germany. Authorities aim at deriving strategies for larger territorial areas, arguing that ‘the district of Heinsberg portrays the nationwide occurrence of infections in a nutshell’ (Ärzteblatt 2020).

However, in the corona crisis some of the limitations of thinking of crisis in territorial units and instruments of territorial scaling also come to light. Even though the corona virus crosses geographical and territorial boundaries, the virus does not spread homogeneously in space. As in many other countries, the shutdown of public life in Germany is a nationwide strategy (with variances across different federal states). This also means that more and less affected areas are treated the same way. When discussing potential strategies for the time after the shutdown these questions of territorial heterogeneity of the outbreak increasingly come to the fore. Regionally differentiated strategies are now discussed. More generally, this can be interpreted as a balancing of upscaling and downscaling, meaning that central (nationwide) measures (or the revocation of such) can be accompanied by punctual local intensification of measures.

On a more general level, the corona crisis demonstrates the interplay of territory and scale by pointing to strategies, limitations and challenges of upscaling and downscaling processes (see also Boin *et al*. 2005, on upscaling). Also fundamental differences occur between centralistic and federal states. Determining *the right scale*, activation of respective structures when necessary and flexible adjustments in territorial scaling are central issues of crisis management. Yet, as observed in other cases, the transgressive forces driving the corona crisis requires complex settings of multi‐level governance that includes several scales and political sectors (Bundy *et al*. 2017). Another interesting question is whether or not the scale of the crisis and the scale of crisis response always have to be congruent for most effective crisis management.

### Territory‐place‐network: ‘social distancing’ policies

The spreading of the corona virus takes place from human to human being. Without changes in the social behaviour, every infected person in average spreads the virus to 2–3 other people. Therefore, most national authorities have enforced so‐called ‘social distancing’ policies. The aim is to reduce the ratio of infection, in the ideal case below 1 (which means in the long run the epidemic will run out because then, statistically, each infected person infects less than one other person). A chain of infections can be interpreted as a network (Kuebart & Stabler, 2020), with every infected person representing a node and every infection from person to person representing a tie. In the terminology of structural network analysis, decreasing infection rates lead to decreasing network connectivity.

From a geographically informed perspective on proximity and distance, the term ‘social distancing’ is a bit misleading, as it suggests that social contacts should be avoided. In fact, rather on the contrary, social distancing encompasses a set of behavioural regulations that seek to allow social contacts, yet in a way that minimises physical proximity and thus promises to disrupt the chain of infections. ‘Social proximity’ thus enables physical distancing since through grown and trusted relationships, familiar face‐to‐face interaction in physical co‐presence can partly be substituted by online media and the like (Boschma 2005).

Social distancing policies do not only address interaction between people, they also include the spatial setting in which interaction occurs. The discourse on super‐spreaders, for instance, focuses not only particular persons who spread the virus at disproportionally high rates, but almost always also includes particular types of places, where the infective encounters took place. Hence, social distancing policies almost always are place sensitive and frequently entail the closure of the respective venues (night clubs, pubs, sports stadiums, concert halls, even playgrounds).

Finally, social distancing policies are enforced on a territorial level, most typically by the national states. However, different territorial approaches co‐existed. While today most countries pursue social distancing policies, not all did so or did not from the very beginning. For instance, Sweden, the Netherlands and the UK preferred another approach of isolating only the most vulnerable individuals while the rest of the population can face the risk of infection in order to reach ‘herd immunity’ sooner rather than later. Other countries, especially in Asia, concentrated on infected persons and followed the strategy of preemptive mass‐testing to identify infections early on and of isolating infected persons from the rest of the population. Territorial differences in terms of crisis response are also known from other crises. Regarding the H1N1 pandemic 2009 (better known as swine flu), Baekkeskov and Öberg (2017) analysed different vaccination policies of Denmark and Sweden, each supported by the dominant national expert opinions. While Sweden followed the approach of vaccinating large parts of its population, Denmark decided to recommend vaccination for risk groups only. Both policies were supported by the majority of expert opinions reported in the respective national mass media. Their findings emphasise that territorial differences in policy strategies are reflected by public discourses on the crisis in the territories.

Even though the general direction of social distancing policies is similar everywhere, there is much variation in detail between territories. For instance, Italy and Spain sought to decrease the amount of social contacts by imposing a lockdown, hence people are no longer allowed to leave their private homes apart from buying food or for health services. In Germany, in contrast, authorities declared a prohibition of social interaction. German citizens are thus still allowed to leave their homes, as long as they follow the commandments of keeping a minimum distance to other citizens of 1.5 metres and seeking only the company of members who live in the same household or at most one other person. As a federal state, however, Germany resembles a fragmented patchwork of territories with slightly different rules and approaches (see above).

Another set of interesting territorial differences in social distancing policies occur in the attribution of surgical masks in public spaces. In Japan, for instance, ‘Mask‐wearing since the 2000s … became a civic duty of those who sneeze and cough not to be a source infection, while for the healthy general public, mask‐wearing embodies neoliberal ethics of being self‐caring and self‐responsible to one’s health’ (Horii 2014). While Japan is the internationally most well‐known example, similar practices can be observed in other countries, especially in East Asia, as well. In the European context, by contrast, the same practice has been widely dismissed by public opinion until very recently. Here, the wearing of surgical equipment is seen as part of a professional practice that is little useful when used inappropriately by laypersons and outside of the professional setting. Therefore, surgical masks played a major role in some national policies in the East‐Asian context while they have been ignored in most Western contexts. However, the perception of masks has shifted quickly recently, as Austria exemplifies, whose government decreed at the beginning of April 2020 the duty of wearing a mask when entering a supermarket. In Germany, the national government recommended the use of masks in the public space in mid‐April. One by one, the Federal states governments did not only take up this recommendation but, similar to Austria, even tighten the rule by declaring the obligation of wearing masks in retail stores or in public transport.

## Opportunities of Crisis Research

In this paper we set out to suggest a conceptually grounded notion of crisis and to explore its geography. The present corona crisis served as an illustrative empirical background to substantiate the analytical spatial dimensions with concrete examples. The term crisis, we suggest with references to contributions from crisis management and organisation studies, encompasses the elements of uncertainty, urgency and threat. Crises are related to societal problems, yet cannot directly be deduced from them. Rather, a crisis becomes only a crisis, if the situation is collectively perceived and declared as a crisis. Moreover, crisis has performative qualities. A crisis diagnosis thus is not primarily a proper description of reality, but a creator of a new reality in which uncertainty, urgency and threat predominate, no matter if decision‐makers like it or not. Right because of the performative nature of crisis diagnoses, the discursive framing of the crisis is a highly contested issue in public debates. It takes place in complex, multi‐stakeholder settings and different interests and worldviews are mobilised. Some stakeholders might even be driven by a strategic interest in further escalating the situation (Löfstedt & Renn 1997).

While crisis management has spent considerable effort to theorise on the temporal aspects of crisis, reference to its spatial aspects remained sparse. Against this background, we suggest that human geography can contribute to inter‐disciplinary research on crisis by unpacking the geographical aspects systematically. We used the TPSN heuristic as suggested by Jessop *et al*. (2008) to delve into the different dimensions of the spatiality of crisis: we explored its territorial dimension, its scalarity, place‐based accounts and the relational spaces of networks. Furthermore, the corona crisis served as a vivid example to illustrate that the TPSN approach is not primarily valuable to disentangle empirical observations and rearrange them along separate dimensions. Rather on the contrary, we used the examples of the recently observable restructuring of supermarket spaces, of flexible re‐scaling of crisis response policies and of social distancing policies to demonstrate that it seems much more promising to scrutinise the multiple forms of interaction and overlap of several spatial dimensions in the same empirical observation.

What is the particular contribution of a conceptually informed, geographical understanding of crisis? We see at least three distinct qualities of such an approach: first, an emerging topic related to the corona crisis is regionally specific response strategies. Here a geographically informed understanding of crisis has much to contribute to the debate. It could support approaches that seek to adapt policies to different regional characteristics (e.g. social distancing policies for urban or rural regions) or to regionally unequally affected areas (e.g. hotspots of the crisis vs. little or no affected areas). Second, and related to the first point, systematically thinking about the geography of crisis can contribute a lot to the question of scalarity in crisis. The corona crisis (as many other crises) demonstrates the challenge of defining the scale of the crisis and respective crisis response strategies (which scale is the right one to (re)act on crises? How to choose the right scale? Does the chosen scale necessarily have to match with a territorial unit?). In fact, due to its transboundary character it evades any single scale and instead calls for complex strategies of multi‐level governance adapted to the institutional idiosyncrasies of different nation states. Third, the corona crisis forces the rapid implementation of several new practices such as avoiding hand contact in supermarkets. Thus, specific places transformed into critical localities, rapidly equipped with special safety infrastructure. The transformation of specific places is observable in our daily lives; however, it cannot fully be understood without references to other similar places. Geography established an analytical understanding of the relations between mobility of practices (supermarket A and supermarket B) and context dependency of practices, enabling a more profound understanding of currently emerging crisis topologies.

The corona crisis will certainly occupy us for a long time. A variety of studies and research projects will surely start in the near future (some already started) in order to reflect on specific aspects of the crisis. Our aim in this paper was to closer investigate the notion of crisis and how a crisis diagnosis changes the present, as well as the view of the past and the future. Crises unfold in time *and space*. The exact geography of a crisis, of course, depends on the empirical case. However, just as thinking about the temporality of crisis, the spatiality of crisis is worth investigating. We made one proposal by drawing on the TPSN framework (Jessop *et al*. 2008) but possible approaches are far from exhausted. We argue for a stronger engagement with ‘crisis’ within human geography since its spatiality is so far kind of an empty space in crisis research.
